# The role of the prostaglandin E2 receptors in vulnerability of oligodendrocyte precursor cells to death

**DOI:** 10.1186/s12974-015-0323-7

**Published:** 2015-05-23

**Authors:** Noel G. Carlson, Satya Bellamkonda, Linda Schmidt, Jonathan Redd, Thomas Huecksteadt, Lauren Marissa Weber, Ethan Davis, Blair Wood, Takayuki Maruyama, John W. Rose

**Affiliations:** Geriatric Research, Education Clinical Center (GRECC), Salt Lake City, USA; Neurovirology Laboratory, VASLCHCS, Salt Lake City, UT USA; Center on Aging, University of Utah, Salt Lake City, UT USA; Brain Institute, University of Utah, Salt Lake City, UT USA; Departments of Neurobiology & Anatomy, University of Utah, Salt Lake City, UT USA; Neuroimmunology and Neurovirology Division, Department of Neurology, University of Utah, Salt Lake City, UT USA; Ono Pharmaceutical Co., Osaka, Japan; Neurovirology Research Laboratory, (151B), VA SLCHCS, 500 Foothill Dr., Salt Lake City, UT 84148 USA

**Keywords:** Oligodendrocyte precursor cells (OPCs), Cyclooxygenase, Excitotoxicity, Prostaglandin E, EP3 receptor

## Abstract

**Background:**

Activity of cyclooxygenase 2 (COX-2) in mouse oligodendrocyte precursor cells (OPCs) modulates vulnerability to excitotoxic challenge. The mechanism by which COX-2 renders OPCs more sensitive to excitotoxicity is not known. In the present study, we examined the hypothesis that OPC excitotoxic death is augmented by COX-2-generated prostaglandin E2 (PGE_2_) acting on specific prostanoid receptors which could contribute to OPC death.

**Methods:**

Dispersed OPC cultures prepared from mice brains were examined for expression of PGE_2_ receptors and the ability to generate PGE_2_ following activation of glutamate receptors with kainic acid (KA). OPC death in cultures was induced by either KA, 3′-O-(Benzoyl) benzoyl ATP (BzATP) (which stimulates the purinergic receptor P2X7), or TNFα, and the effects of EP3 receptor agonists and antagonists on OPC viability were examined.

**Results:**

Stimulation of OPC cultures with KA resulted in nearly a twofold increase in PGE_2_. OPCs expressed all four PGE receptors (EP1–EP4) as indicated by immunofluorescence and Western blot analyses; however, EP3 was the most abundantly expressed. The EP3 receptor was identified as a candidate contributing to OPC excitotoxic death based on pharmacological evidence. Treatment of OPCs with an EP1/EP3 agonist 17 phenyl-trinor PGE_2_ reversed protection from a COX-2 inhibitor while inhibition of EP3 receptor protected OPCs from excitotoxicity. Inhibition with an EP1 antagonist had no effect on OPC excitotoxic death. Moreover, inhibition of EP3 was protective against toxic stimulation with KA, BzATP, or TNFα.

**Conclusion:**

Therefore, inhibitors of the EP3 receptor appear to enhance survival of OPCs following toxic challenge and may help facilitate remyelination.

## Introduction

Cyclooxygenase (COX) catalyzes the rate-limiting step in the synthesis of prostanoids from arachidonic acid [1]. Two isoforms of COX have been identified: a constitutive form designated COX-1 and an inducible form, COX-2 [2]. In the central nervous system (CNS), COX-2 expression is increased in neurons in response glutamate receptor activation [3, 4]. COX inhibitors termed non-steroidal anti-inflammatory drugs (NSAIDs) that are specific to COX-2 promote neuronal survival *in vitro* [2, 3] and *in vivo* [4] following induction of glutamate-receptor-mediated excitotoxic death.

Genetic evidence also indicates a role for COX-2 in excitotoxicity. Transgenic mice that over-express neuronal COX-2 are more susceptible to excitotoxicity [5] and age-associated neuronal loss [6]. In contrast, COX-2 null (knockout) mice exhibit less neuronal death following ischemia or challenge with NMDA [7]. Therefore, pharmacological and genetic evidence reveals that COX-2 expression and activity contributes to neuronal excitotoxic cell death. Using this analogy as a framework for the role of COX-2 in death of oligodendrocytes (OLs), we showed that COX-2 is induced in OLs and OPCs following glutamate receptor (GluR) activation and renders these cells more susceptible to excitotoxic death [8]. We also have shown that COX-2 is expressed in dying OLs at the onset of demyelination in Theiler’s Murine Encephalomyelitis Virus (TMEV) model of multiple sclerosis (MS) [9] and in dying OLs in MS lesions [8]. Additional studies have shown that COX-2 also contributes to OL vulnerability in the cuprizone model of demyelination [10]. These studies suggest that COX-2 may have an important role in demyelinating diseases like MS.

Studies with COX-2 inhibitors in animal models of MS also support a role for COX-2 as a contributor to disease pathology [11, 12]. Two groups have reported that administration of COX-2 inhibitors in experimental autoimmune encephalomyelitis (EAE) diminished the severity and incidence of disease and decreased demyelination and inflammation [11, 12]. In both cases, the therapeutic effects in EAE were only observed when the COX-2 inhibitors were initiated immediately after immunization and maintained throughout the course of the study. In these cases, COX-2 inhibition in the induction phase of EAE was due in part to immunomodulatory effects resulting from suppression of T-cell signaling through interleukin-12 (IL-12) [11]. In addition, our group has shown that COX-2 inhibitors reduce demyelination in the TMEV model of MS [8]. A recent study by Esaki et al. examined the role of PGE_2_ receptor signaling in EAE and identified a role for EP2 and EP4 in peripheral immune response and increase of blood–brain barrier permeability in the initiation and progression of monophasic EAE using global knockouts of PG receptors [13]. However, their studies do not address the potential contribution of PG receptors towards modulation of OPC viability and remyelination.

In EAE, excitotoxicity and axonal damage appear to contribute to the pathology of the disease, since α-amino-3-hydroxy-5-methyl-4-isoxazolepropionic acid (AMPA) antagonists of GluRs can ameliorate the neurological deficits associated with the progression of the disease [14]. This affect may in part be due to injury of OLs and OPCs which express GluRs of the AMPA and kainate classes and are also susceptible to glutamate-mediated excitotoxicity [15]. This may be particularly important for OPCs since the susceptibility of OPCs to injury within the MS lesion environment can be a major limitation to remyelination in MS [16].

In this study, we examined whether prostanoids (PGs) such as PGE_2_ and their receptors contribute to excitotoxic death of OPCs. We examined whether PGE_2_ was made by OPCs and whether activation of specific PGE_2_ receptors contributes to the vulnerability of OPCs.

## Methods

### Materials

Tissue culture media and reagents along with the kainic acid and 3′-O-(Benzoyl) benzoyl ATP (BzATP) were purchased from Sigma Chemical Company (Saint Louis, MO). Recombinant mouse TNFα was purchased from R&D systems (Minneapolis, MN). Fetal bovine serum and horse serum were purchased from Hyclone (Logan, UT). All the COX-2 inhibitors (CAY 10452, NS398, and CAY 10404) and the EP2 agonist butaprost were purchased from Cayman Chemical Company (Ann Arbor, MI). The EP3 antagonist ONO-AE5-599 was provided by Ono Pharmaceuticals.

### Immunofluorescence confocal microscopy

Immunoreactivity was assessed with primary antibodies to mouse antigens that included anti-EP1, EP2, EP3, and EP4 (Cayman Chemicals, Ann Arbor, MI). These antibodies have been shown to have high specificity towards each EP receptor with little to no detectable cross-reactivity between different EP receptors [17]. Antibodies to Olig 1 were from Abcam (Cambridge, England). Primary antibodies were used at dilutions recommended by the manufacturers. Secondary fluorochrome antibodies for mouse were donkey fluorescein isothiocyanate (FITC)-conjugated anti-rabbit and Cy5-conjugated anti-mouse/rat (Jackson ImmunoResearch laboratories, West Grove, PA) and donkey FITC anti-goat, Cy5 anti-mouse, and C3 anti-rabbit. Secondary antibodies were used at concentrations recommended by the manufacturers. The primary antibodies were incubated overnight in a humidified chamber at 4 °C. Secondary antibodies were added for 1 h at room temperature. Background staining was assessed with negative controls consisting of 20 μg/ml normal mouse/rat serum and 30 μg/ml normal rabbit serum. After staining with antibodies then propidium Iodide (PI), coverslips were mounted onto the samples using ProLong Gold anti-fade mounting media (Molecular Probes Inc., Eugene, OR). The Personal Confocal Microscopy PCM-2000 (NIKON, Melville, NY) with argon, green, and red HeNe lasers was used to acquire images from the three different fluorochromes. The Simple Personal Confocal Image program (PCI, Compix, Cranberry Township, PA) was used to acquire digital images for the three different image channels. The FITC label was detected with the argon laser at 488 nm, Cy5 with the red argon laser at 633 nm, and Cy3 was visualized with the green HeNe laser at 563 nm. Tissues were individually scanned with each respective laser filter. All images were acquired using the multi-focal program (z-focus) to create a stereopsis image. The three different images were merged together to acquire the final three-colored image, and the PI image was converted to blue color during merge.

### Dispersed oligodendrocyte cultures and toxicity assay

All aspects of animal handling and care were conducted with local Institutional Animal Care and Use Committee (IACUC) approval in an Association for Assessment and Accreditation of Laboratory Animal Care (AAALAC)-approved facility (The Veterans Affairs Salt Lake City Health Care System Veterinary Medical Unit). Dispersed oligodendrocyte cultures were prepared from P1 mouse pups as in our earlier study [8] which was originally performed as described in [18]. Contaminating microglia were depleted from the OPCs by mixing the cultures with magnetic particles containing anti-CD64 antibodies (BD Biosciences, San Jose, CA) and placing the culture tube next to a magnet while the OPC culture suspension is removed. OPCs were plated in 96 well plates and photographed using phase contrast microscopy prior to treatment with kainic acid. Purity of the cell culture was verified by staining a sister well with the OPC marker Olig 1. The same fields were photographed 24, 48, or 144 h after treatment with agents that induce OPC death. Each treatment group typically contained 200–400 cells. For toxicity experiments, oligodendrocytes were scored as living if the cell did not stain with PI. The percent survival was calculated by dividing the number of live cells not stained with PI observed after kainic acid (KA) treatment divided by the number of cells present in the same field prior to KA treatment. Three or more fields were captured (at a magnification of ×20) for each treatment group. This assay has been used to assess OPC survival [8] and is similar to our previous published assays to determine neuronal survival following excitotoxicity [3, 19]. The percent survival was calculated as percent control relative to the survival observed with no KA treatment. Background death was typically less than 25 %.

### Measurement of prostaglandin E2 from cultured OPCs

The media on the OPC cultures was replaced with fresh pre-warmed media containing either KA or vehicle. After a 4-h incubation at 37 °C, the media was removed, immediately spun at 2000×*g* (to remove any cells or debris), and frozen at −80 °C for subsequent analyses. The concentration of KA used normally would yield a high amount of toxicity (80 % cells killed) after 24 h of treatment, but no cell death was apparent at this time. Frozen media samples were analyzed using PGE_2_ enzyme-linked immunosorbent assay (ELISA) kit from Cayman Chemical Company (Ann Arbor, MI). This assay can detect concentrations of PGE_2_ to as low as 15 pg/ml.

### Western blot analyses of EP receptors

OPCs from cultures were rinsed in PBS and subsequently lysed in SDS sample buffer containing protease inhibitors (cOmplete ULTRA®-protease inhibitor, Roche, Mannheim, Germany). The samples were fractionated on a 10 % acrylamide gel by electrophoresis, then electro blot transferred to nitrocellulose and probed with the same rabbit antibodies to EP1–EP4 (used at a dilution of 1:200) along with mouse anti-oligodendrocyte-specific protein (OSP) (used at a dilution of 1:400) to normalize for loading. The blot was washed and then probed with anti-rabbit and anti-mouse secondary antibodies (Licor Biotechnology, Lincoln, NE) (used at a dilution of 1:5000) and visualized using the Li-COR imaging system. Densitometry scanning of the Western blot image files was performed using image J to quantify the relative intensity of each EP receptor, and this was normalized to OSP for each lane. The relative amounts were quantified from triplicate gels and assessed for statistical differences using ANOVA, Tukey-Kramer multiple comparison test.

### Real-time PCR of EP3 transcripts

Total cellular RNA was isolated from cultured OPCs using RNeasy protocols (Qiagen, Limburg, Netherlands). First-strand cDNA was reverse-transcribed from 1.0 μg of total RNA using a High Capacity cDNA Reverse Transcription Kit (Life Technologies, Carlsbad, CA). The EP3 splice variants and glyceraldehyde 3-phosphate dehydrogenase (GAPDH) were quantified using primer pairs listed below using an ABI 7500 Real-Time PCR System (Life Technologies, Carlsbad, CA). cDNA was mixed with Power SYBR® Green PCR Master Mix (Life Technologies, Carlsbad, CA) and the appropriate primers for the gene of interest. We used the comparative cycle threshold (CT) method (2^∆∆CT^) to calculate relative gene expression under experimental and control conditions normalized to GAPDH. The results are expressed as a fold change over-expression relative to the beta isoform which was the least abundant isoform [20]. The primers from Zhang et al. [21] for each splice variant are listed below along with the size of the PCR product.

EP3alpha: sense 5′-GGATCATGTGTGTGCTGTCC-3′ and

antisense 5′-GCAGAACTTCCGAAGAAGGA 3′, 218 bp;

EP3beta: sense 5′-TGAACAACCTGAAGTGGACTTTC-3′ and

antisense 5′-ATTCTCAGACCCAGGGAAACAGG-3′, 60 bp;

EP3gamma: sense 5′-TTCGCTGAACCAGATCTTGGATC-3′ and

antisense 5′-TAGACAATGAGATGGCCTGCCCT-3′, 136 bp,

The primers for GAPDH were from Nozaki et al. [22] and are listed below.

GAPDH sense 5′-TGGCAAAGTGGAGATTGTTGCC-3′ and

GAPDH antisense 5′AAGATGGTGATGGGCTTCCCG-3′, 156 bp.

### Statistical analysis

Data were analyzed using InStat3, a statistical software package (graph pad Prism, San Diego, CA). Assessment of significance of cell death between groups and differences in relative EP3 splice variants was done using ANOVA Tukey-Kramer multiple comparisons test or pair wise using the Student *t*-test. All comparisons satisfied the Kolmogorov and Smirnov assumption test for Gaussian distributions, thus allowing parametric analyses.

## Results

### GluR activation stimulates synthesis of PGE_2_

**Fig. 1:**
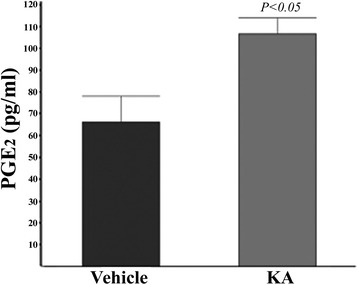
OLs produce PGE_2_ in response to GluR activation. Media from OPCs treated with KA (10 mM for 4 h) or control (vehicle) were analyzed for PGE_2_ by ELISA (see the “Methods” section). No cell death was observed at 4 h treatment (a 20-h exposure was required to produce death)

In order to identify how COX-2 may contribute to OL viability, we first asked whether any of the major prostanoids are synthesized in response to GluR activation. We initially examined PGE_2_ because this prostanoid is important in modulating neuronal viability in response to excitotoxic stimuli [3, 19, 23–30]. OPC cultures were treated with kainic acid (KA) at a concentration that would stimulate approximately 80 % death 20 h after treatment, and the media was removed 4 h later. At this time, there was no evidence of cell death. The media was analyzed by ELISA for the amount of PGE_2_ in vehicle and KA-treated cultures. As seen in Fig. 1, there was nearly a twofold increase in the amount of PGE_2_ in the media from KA-treated cells compared to vehicle-treated controls. The concentration of PGE_2_ in the KA-treated cultures was approximately 100 pg/ml (roughly 0.3 nM). This concentration of PGE_2_ is 1 to 2 orders of magnitude below the dissociation constant for the PGE_2_ receptors [30]. However, locally higher concentrations are likely present near the site of synthesis in the cells and are of physiologic significance.

### Expression of PGE_2_ receptors in OPCs

**Fig. 2:**
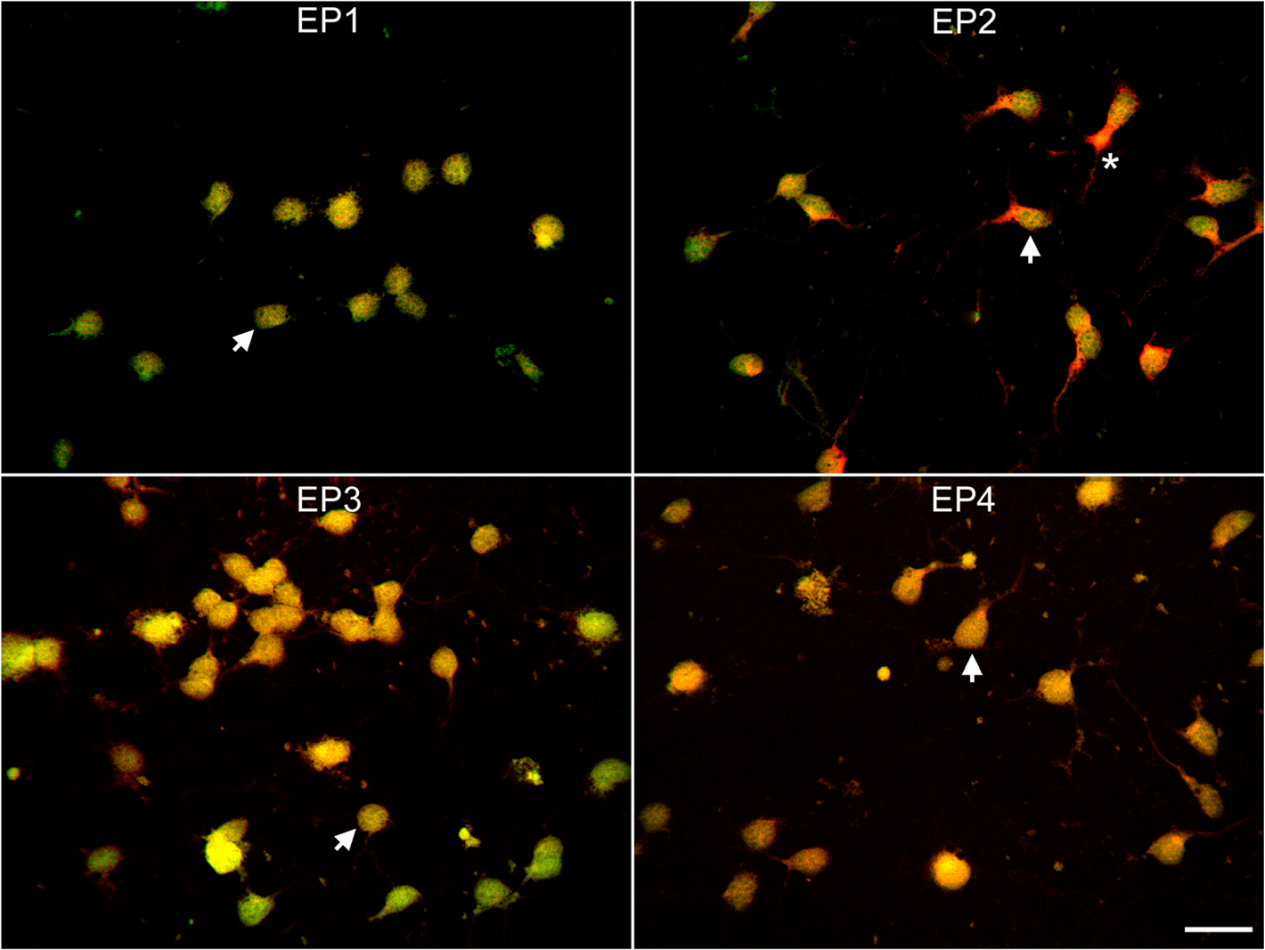
Expression of EP receptors in purified oligodendrocytes. Purified OPC cultures were stained with the oligodendrocyte marker Olig 1 (green) and EP1 through EP4 (red) as indicated. Co-expression appears yellow, and examples are indicated with *arrows*. The *asterisk* indicates an example where immunoreactivity for EP2 is present predominantly in the cell processes. The bar in the lower corner is 20 μm

**Fig. 3:**
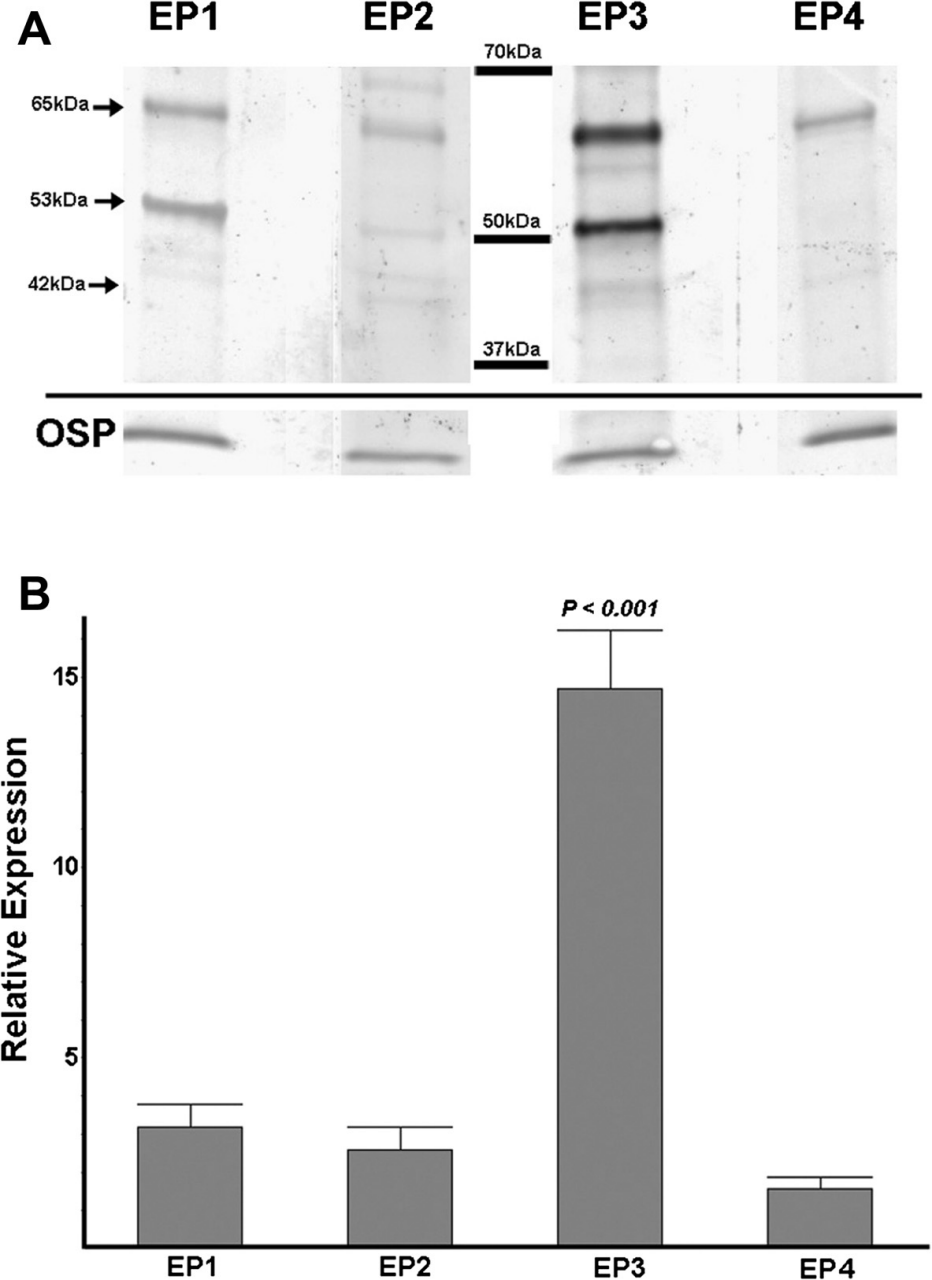
Western blot of EP receptors. **a** OPCs were analyzed by Western blot for expression of EP1–EP4 receptors. Expression of oligodendrocyte-specific protein (OSP) at 22 KDa was examined to control for sample loading. Expected bands at 42, 53, and 65 kDa were observed for the EP receptors. **b** The digital images from three replicate blots were quantified for the intensity of the EP receptor bands and normalized to the intensity of OSP. The average values were plotted for EP1–EP4. EP3 was significantly higher than all of the other three species (*P <* 0.001 Tukey-Kramer multiple comparison test ANOVA)

**Fig. 4:**
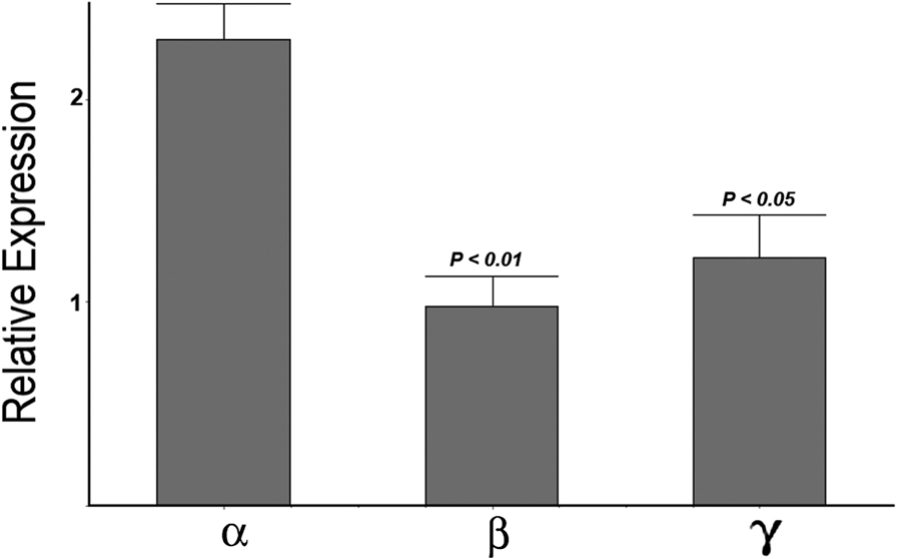
Expression of EP3 splice variants. Real-time PCR was performed on cDNA generated from OPC RNA with primer pairs specific to each of the three EP3 splice variants. GAPDH was used as an internal standard for comparison for each splice variant (see the Methods section). The alpha variant was significantly more abundant than the beta and gamma variants

We next examined which of the four PGE_2_ receptor subtypes (EP1–EP4) were expressed in cultured OPCs. Confocal immunofluorescence analyses of cultured OPCs with antibodies to all four EP receptors and the OPC marker Olig 1 detected expression of all four receptor subtypes (EP1–EP4) to varying degrees (Fig. 2). Expression of each subunit was confirmed using Western blot analyses, and immunoreactive bands to each subunit at the predicted molecular weights were detected (Fig. 3). EP3 was significantly higher at levels that were nearly fivefold greater than the other EP receptors. The EP3 receptor subunit has been reported to exist in three potential splice variants in mice [31] which may impart different properties to the receptor and couple to different G proteins [31]. EP3 transcripts from OPCs were analyzed by real-time PCR to assess the relative amount of expression of each variant. As seen in Fig. 4, all three splice variants of EP3 were expressed in OPCs. However, the α variant was significantly more abundant than either the β or γ variants.

### EP receptor contribution to OPC viability

**Fig. 5:**
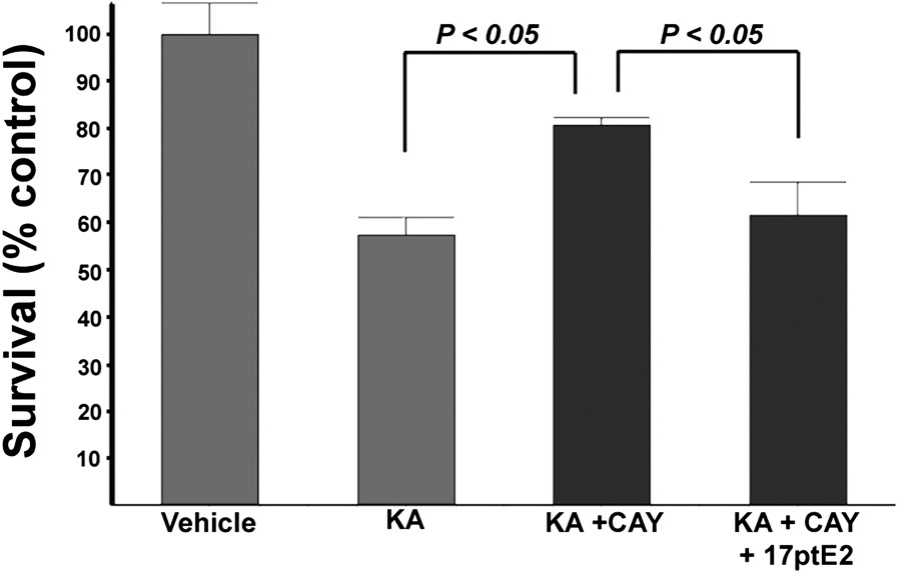
The EP1/EP3-specific agonist 17ptE2 reverses the protective effect of the COX-2 inhibitor CAY10404. Dispersed OPCs treated with KA and the COX-2 inhibitor CAY (10 μM) resulted in a significant increase in surviving OPCs. When 17ptE2 (1 μM) was included with CAY, the protective effect was lost. Treatment with either CAY or 17ptE2 alone or in combination in the absence of KA was not toxic to OPCs (data not shown). In contrast to the other experiments where survival was assessed 48–144 h after addition of KA, in this experiment, survival was assessed 24 h after KA because of concerns of the chemical and metabolic stability of 17ptE2 with longer incubations. Significance was observed using ANOVA analyses. This result is representative of three different experiments

In our earlier studies, we demonstrated that COX-2 expression in OPCs contributes to excitotoxic vulnerability [8]. We showed that a pharmacological blockade of COX-2 or reduced expression by genetic means can render OPCs less vulnerable to excitotoxic death [8]. Since all four EP receptors are expressed in OPCs, we then examined whether any of these receptors may be in part responsible for the contribution of COX-2 towards excitotoxic death of OPCs. We initially determined whether activation of specific EP receptors could reverse the protective effect of a COX-2 inhibitor. We observed that stimulation of excitotoxic death by KA can be diminished by treatment with a COX-2-specific inhibitor CAY10404 (Fig. 5). However, when we included the metabolically stable (and chemically stable) agonist of EP1 and EP3 receptors 17-phenyl-trinor PGE_2_ (17ptE2) [32] with the COX-2 inhibitor, the protective effect of the COX-2 inhibitor was abolished (Fig. 5). No death was observed in the absence of KA when cultures were treated with 17ptE2 with or without CAY10404 (data not shown). These results indicate that activation of an EP receptor can reverse the protective effect of a COX-2 inhibitor.

### Contribution of EP receptors to excitotoxic death

**Fig. 6:**
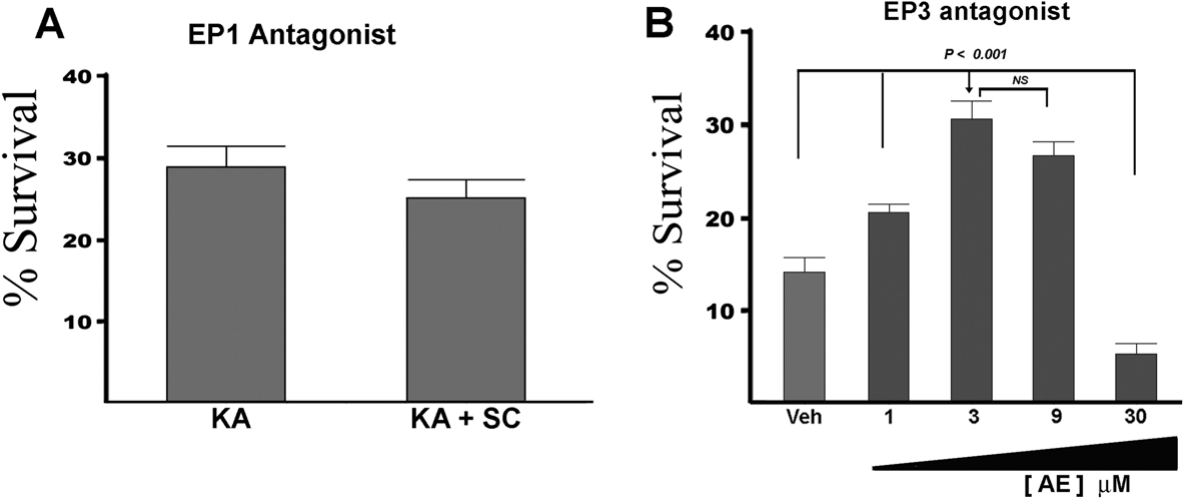
Protective properties of EP1 and EP3 antagonists. **a** The EP1 antagonist SC51089 (SC) (1 μM) showed no protection of OPCs against excitotoxic death. **b** However, the EP3 antagonist ONO-AE5-599 (AE) shows varying amounts of protection across a range of concentrations ranging from 1–30 μM. The lowest concentration of AE that gave the greatest amount of protection was observed at 3 μM. Dispersed OPCs were treated with KA in the presence or absence of SC or AE and analyzed for survival 144 h later. Similar levels of protection of each agent were also observed at 24 and 48 h after KA (not shown). As a positive control for efficacy of SC, parallel treatment of cortical neurons with 1 μM SC conferred neuroprotection of cultured neurons against NMDA-induced excitotoxicity as observed in our previous study [19] (data not shown). The results shown in this figure are representative of four different experiments

The EP receptor agonist 17ptE2 is highly specific for both EP1 and EP3, making these receptors possible candidates for contributors to excitotoxic death of OPCs. In order to test directly which of the receptors contributes to the excitotoxic death of OPCs, we then examined whether specific antagonists of these receptors could have protective effects. As seen in Fig. 6a, the EP1-specific antagonist (SC51089) [32] conferred no protective effect against KA-induced excitotoxicity. However, the EP3-specific antagonist (ONO-AE5-599) [33] conferred a protective effect against KA-induced excitotoxicity across a range of concentrations with the maximal protection at 3 μM (Fig. 6b).

### Antagonism of EP3 also protects against excitotoxicity following activation of the purinergic receptor P2X7 and treatment with TNFα

**Fig. 7:**
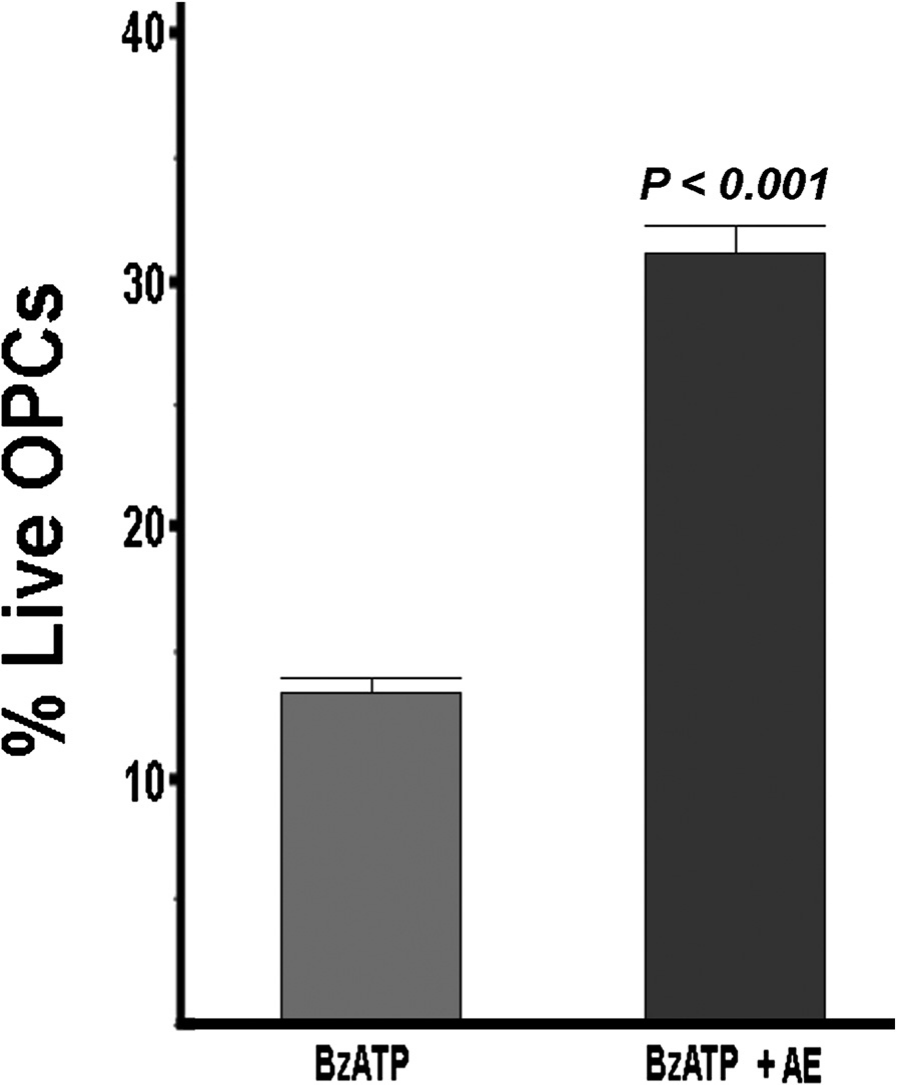
The EP3 antagonist ONO-AE5-599 (AE) protects OPCs against excitotoxic death stimulated by BzATP (a specific agonist of P2X7). Dispersed OPCs were treated with BzATP (500 μM) in the presence or absence of AE5-599 (3 μM). Surviving cells were counted 48 h after treatment with BzATP. There was a greater than a twofold significant increase (*P* < 0.0001 by Student *t*-test) in surviving OPCs. This result is representative of five different experiments

In order to determine if protection resulting from inhibition of the EP3 receptor was only confined to GluR-mediated excitotoxicity, we asked whether this prostaglandin receptor could be involved with other stimulators of OPC death. Activation of the ionotropic purinergic receptor P2X7 is also a potent contributor to excitotoxic death of OPCs [34]. As seen in Fig. 7, stimulation of cultured OPCs with the P2X7-specific agonist BzATP results in cell death. However, when the EP3-specific antagonist is included, significant protection against BzATP-induced cell death is observed. These results establish that EP3 is a significant contributor to excitotoxic death of OPCs.

**Fig. 8:**
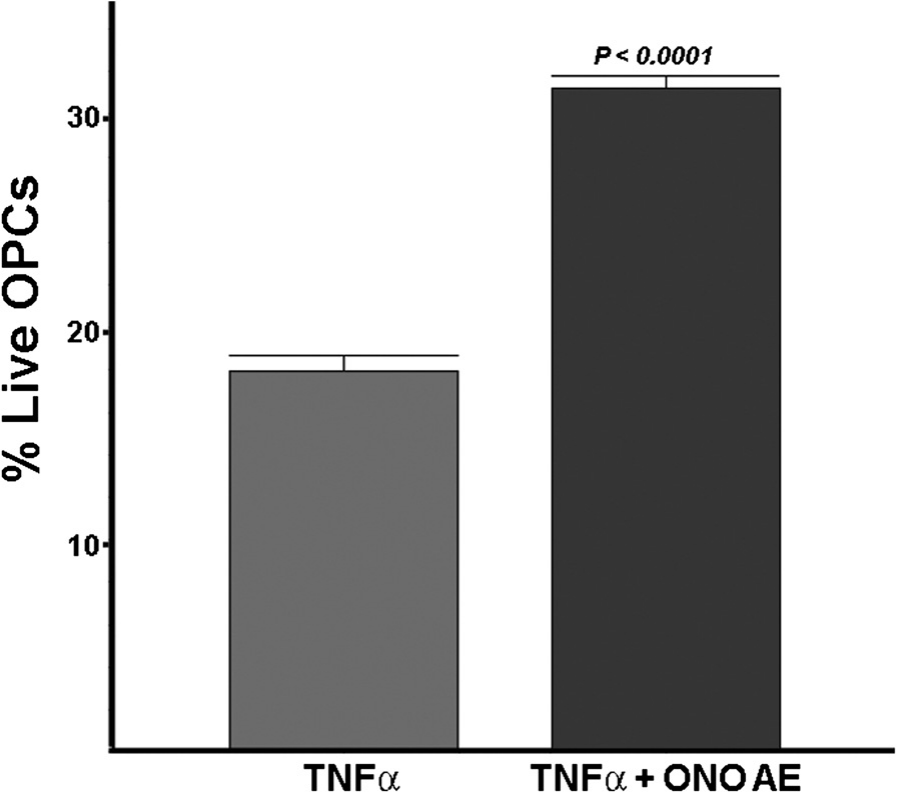
Protection of OPCs against TNFα-induced death. OPCs were treated with 400-ng recombinant mouse TNFα in the presence or absence of the EP3 antagonist ONO-AE5-599 (1 μM), and death was assessed 48 h later. There was a nearly twofold significant increase (*P* < 0.0001 by Student *t*-test) in surviving OPCs. This result is representative of five different experiments

Another potent stimulator of OPC death is the cytokine TNFα [16]. As seen in Fig. 8, treatment of OPC cultures with 400 μg/ml recombinant mouse TNFα resulted in substantial death of OPCs, which was significantly attenuated when the specific EP3 antagonist was present. These findings identify that EP3 may play a broader role beyond just excitotoxic stimuli for contributing to vulnerability of OPCs to injury.

### Expression of EP3 in OLs in MS lesions

**Fig. 9:**
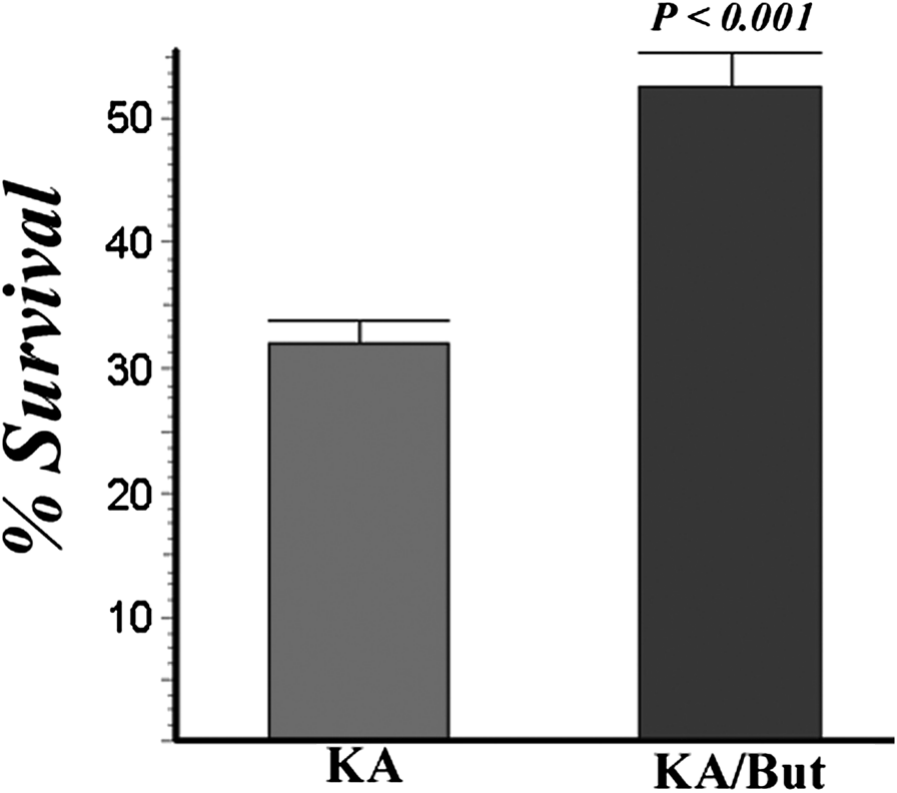
The EP2 agonist butaprost (But) protects OPCs from KA-induced toxicity. OPC survival was assessed 48 h following treatment of OPCs with KA in the presence or absence of butaprost (500 nM). This graph represents the combined data from four independent experiments. Significant protection (Student *t*-test) was observed for Butaprost

To show relevance of these experimental studies to human disease, we examined spinal cord plaques from MS samples to determine whether EP3 was expressed in OLs. As seen in Fig. 9, a representative image of immunofluorescence analyses of tissue from an MS lesion indicated that EP3 was expressed in cells along with the OL marker CNPase. In contrast, EP3 was not detected in OLs from control (non-MS) tissue. Further analyses of one other MS patient yielded similar results of EP3 expression in OLs (data not shown). These findings are consistent with a potential role for EP3 in influencing survival of OLs.

### Protection of OPCs by activation of the EP2 receptor

In neurons, PGE_2_ receptors such as EP2 can contribute to increased viability following excitotoxic challenge [27]. We next examined whether activation of EP2 in OPCs could be protective against excitotoxicity as is seen in neurons [27]. Treatment of OPCs with the EP2-specific agonist butaprost increased the survival of OPCs following excitotoxic challenge with either KA or BzATP (Fig. 9). These findings show that EP2 can contribute to the viability of OPCs.

## Discussion

In this study, we examined how COX-2 could contribute to the excitotoxic vulnerability of OPCs through stimulation of components of the downstream mediator PGE_2_. We identified PGE_2_ and its receptor EP3 as a major contributor to excitotoxic death of OPCs based on three major lines of evidence: (1) activation of GluRs in OPCs stimulates the synthesis of PGE_2_ in culture, (2) activation of EP3 reverses the protective effect of a COX-2 inhibitor, and (3) inhibition of EP3 is protective against both glutamatergic and purinergic excitotoxic stimuli as well as inflammatory mediators such as TNFα. We also identified that activation of the prostaglandin EP2 receptor renders OPCs less vulnerable to excitotoxicity.

These results suggest that the contributions of EP receptors to OPC viability largely mirror the same type of effects seen in neurons where EP3 can contribute to excitotoxic death [30] and EP2 can have protective effects [27]. However, in contrast to neurons where EP1 appears to be a major contributor to excitotoxic death, we observed no contribution of EP1 to the excitotoxic death of OPCs. It is not clear why EP1 does not appear to play a similar role in OPCs, but it is notable that EP1 did not appear to be as abundantly expressed in OPCs as EP3 (see Figs. 2 and 3). Alternatively, OPCs may lack key effector proteins required for the coupling activation of EP1 to second messenger cascades that are known to activate phospholipase C and intracellular calcium increases linked to neuronal death [7].

EP3 is a G-protein-coupled receptor (GPCR) linked to different second messenger pathways that could impart their effects on modulation of OPC viability following a toxic challenge [30, 31]. The major pathway linked to EP3 activation is mediated through the G protein Gi that is coupled to a decrease in cAMP [31]. However, each of the three different splice variants of EP3 (α, β, and γ) can be coupled to either this pathway or an increase of intracellular calcium linked to the IP3 pathway. The γ variant is unique in that it can also be coupled to an increase in cAMP. We identified that all three isoforms are present in cultured OPCs but that the alpha variant was the most abundant species. This result indicates that activation of EP3 in OPCs could be linked to either changes in cAMP or to an increase in intracellular calcium. Subsequent studies should help to identify which signaling pathways contribute to OPC death.

Our results also indicate that activation of EP2 on OPCs by butaprost is protective against an excitotoxic challenge. Butaprost can also interact with the IP receptor [35], but since we could not detect IP expression in our OPC cultures (data not shown), butaprost is likely acting specifically on the EP2 receptor in our experiments. In other recent studies, EP2 activation may not necessarily be protective against other insults to OLs. An antagonist with modest specificity towards EP2 prevented OL apoptosis in the cuprizone model of demyelination [10]. The discrepancy with our result could be due to the following: (1) differences in OPC and OL death pathways between excitotoxicity and cuprizone-mediated death, (2) the drug that was used may have inhibited other PG receptors other than EP2, or (3) inhibition of EP2 on other cells, such as microglia, may have contributed to OPC protection. It is important to note that another receptor antagonist with high specificity towards EP2 was recently shown to have neuroprotective effects following status epilepticus by acting on microglia [36].

Our results have important ramifications with respect to demyelinating diseases such as MS. It has recently been shown that susceptibility of OPCs within the MS lesion environment can be a major limitation to remyelination in MS [16]. As such, therapies that increase OPC viability could be valuable for increasing OPC number and promoting remyelination which in turn could preserve axons and help limit the progression of disease. We know from our previous work that inhibition of COX-2 has a net effect of increasing OPC viability. However, COX-2 inhibition may not be optimal for promoting OPC survival as suggested by our findings demonstrating that PGE_2_ was able to have both detrimental actions through EP3 and beneficial effects through EP2. In this case, with respect to OPC viability, targeting downstream receptors may be more beneficial by allowing inhibition of just the detrimental receptor (EP3), while allowing activation of the beneficial receptor (EP2). Furthermore, other prostanoids may also have beneficial effects in MS. For instance, prostacyclin (PGI2) has recently been reported to promote migration of OPCs to demyelinated areas to help promote remyelination [37]. Therefore, targeting the detrimental downstream receptors could have added benefits beyond inhibition of the entire repertoire of PGs synthesized with COX-2 inhibitors.

We found that EP3 contributes to the vulnerability of OPCs to injury across three different toxic challenges. Therefore, EP3 may be a general contributor to OPC vulnerability across a spectrum of insults and provide a rationale for inhibiting EP3 receptors to help promote OPC survival which in turn could lead to increased remyelination. Inhibition of EP3 may also have other affects beyond OPC survival which could be of benefit in MS. For example, global EP3 knockout mice have been reported to have decreased neuronal loss, reduced blood–brain damage, and reduced microglial activation in stroke models [30], all of which could be beneficial in MS. Future *in vivo* studies in demyelinating disease models will help delineate the potential role of EP3 in disease pathogenesis.

We have begun to assess whether EP3 may play a role in MS pathogenesis. In ongoing preliminary studies with MS autopsy CNS tissue, we have found that EP3 was extensively associated with OLs on the edge of a MS lesion and was absent in control spinal cord white matter (data not shown). Immunoreactivity of EP3 in the MS plaque was in a region of the spinal cord without inflammation by conventional histology and contained evidence of ongoing demyelination signified by the presence of MBP fragments which are indicative of myelin injury within a 14-day time period. Since EP3 was present in the active edges of MS plaques, it is consistent that this receptor may also play an important role in demyelination and remyelination in MS. Therefore, EP3 may be a potential target for therapies to limit disease progression in demyelinating diseases including MS.

We have recently begun to assess whether other prostanoids may also play a role similar to PGE_2_. We examined whether KA can stimulate OPCs to synthesize any of the other four major prostanoids. Prostaglandin F2α (PGF2α) was the only other prostanoid that was synthesized following treatment of OPCs with KA (data not shown). In ongoing studies, we have found that an inhibitor of the PGF2α receptor, FP, was also protective against KA-induced death (Carlson et al. manuscript in preparation). Further studies are under way to determine the interaction between EP3 and FP in modulation of OPC viability.

## Conclusions

This study demonstrates that the PGE_2_ receptor EP3 contributes to susceptibility of OPCs to death. These results suggest that inhibitors of EP3 could limit OPC death and may help promote remyelination. EP3 inhibitors may be considered as potential new targets for therapies for MS.

## References

[1] Smith WL, Garavito RM, DeWitt DL (1996). Prostaglandin endoperoxide H synthases (cyclooxygenases)-1 and −2. J Biol Chem..

[2] Hewett SJ, Uliasz TF, Vidwans AS, Hewett JA (2000). Cyclooxygenase-2 contributes to N-methyl-D-aspartate-mediated neuronal cell death in primary cortical cell culture. J Pharmacol Exp Ther..

[3] Carlson NG (2003). Neuroprotection of cultured cortical neurons mediated by the cyclooxygenase-2 inhibitor APHS can be reversed by a prostanoid. J Neurosci Res.

[4] Nogawa S, Zhang F, Ross ME, Iadecola C (1997). Cyclooxygenase-2 gene expression in neurons contributes to ischemic brain damage. J Neurosci..

[5] Kelley KA, Ho L, Winger D, Freire-Moar J, Borelli CB, Aisen PS (1999). Potentiation of excitotoxicity in transgenic mice overexpressing neuronal cyclooxygenase-2. Am J Pathol..

[6] Andreasson KI, Savonenko A, Vidensky S, Goellner JJ, Zhang Y, Shaffer A (2001). Age dependent cognitive deficits and neuronal apoptosis in cyclooxygenase-2 transgenic mice. J Neurosci..

[7] Iadecola C, Niwa K, Nogawa S, Zhao X, Nagayama M, Araki E (2001). Reduced susceptibility to ischemic brain injury and N-methyl-D- aspartate-mediated neurotoxicity in cyclooxygenase-2-deficient mice. Proc Natl Acad Sci U S A..

[8] Carlson NG, Rojas MA, Redd JW, Tang P, Wood B, Hill KE (2010). Cyclooxygenase-2 expression in oligodendrocytes increases sensitivity to excitotoxic death. J Neuroinflammation..

[9] Carlson NG, Hill KE, Tsunoda I, Fujinami RS, Rose JW (2006). The pathologic role for COX-2 in apoptotic oligodendrocytes in virus induced demyelinating disease: implications for multiple sclerosis. J Neuroimmunol.

[10] Palumbo S, Toscano CD, Parente L, Weigert R, Bosetti F (2012). The cyclooxygenase-2 pathway via the PGE2 EP2 receptor contributes to oligodendrocytes apoptosis in cuprizone-induced demyelination. J Neurochem..

[11] Muthian G, Raikwar HP, Johnson C, Rajasingh J, Kalgutkar A, Marnett LJ (2006). COX-2 inhibitors modulate IL-12 signaling through JAK-STAT pathway leading to Th1 response in experimental allergic encephalomyelitis. J Clin Immunol.

[12] Miyamoto K, Miyake S, Mizuno M, Oka N, Kusunoki S, Yamamura T (2006). Selective COX-2 inhibitor celecoxib prevents experimental autoimmune encephalomyelitis through COX-2-independent pathway. Brain..

[13] Esaki Y, Li Y, Sakata D, Yao C, Segi-Nishida E, Matsouka T (2010). Dual roles of PGE2-EP4 signaling in mouse experimental autoimmune encephalomyelitis. Proc Natl Acad Sci U S A..

[14] Pitt D, Werner P, Raine CS (2000). Glutamate excitotoxicity in a model of multiple sclerosis. Nat Med..

[15] Verkhratsky A, Steinhauser C (2000). Ion channels in glial cells. Brain Res Rev.

[16] Cui OL, Kuhlmann T, Miron VE, Leong LY, Fang J, Gris P (2013). Oligodendrocyte progenitor cell susceptibility to injury in multiple sclerosis. Am J Pathol..

[17] Akaneya Y, Tsumoto T (2006). Bidirectional trafficking of prostaglandin E2 receptors involved in long-term potentiation in visual cortex. J Neurosci..

[18] Bruce-Keller AJ, Geddes JW, Knapp PE, McFall RW, Keller JN, Holtsberg FW (1999). Anti-death properties of TNF against metabolic poisoning: mitochondrial stabilization by MnSOD. J Neuroimmunol..

[19] Carlson N, Rojas M, Black J-D, Redd J, Hille J, Hill K (2009). Microglial inhibition of neuroprotection by antagonists of EP1 prostaglandin E2 receptor. J Neuroinflammation..

[20] Livak KJ, Schmittgen TD (2001). Analysis of relative gene expression data using real-time quantitative PCR and the 2^−∆∆CT^ method. Methods..

[21] Zhang J, Zou F, Tang J, Zhang Q, Gong Y, Wang Q (2013). Cyclooxygenase-2-derived prostaglandin E2 promotes injury-induced vascular neointimal hyperplasia through the E-prostanoid 3 receptor. Circ Res..

[22] Nozaki I, Lunz JG, Specht S, Stolz DB, Taguchi K, Subbotin VM (2005). Small proline-rich proteins 2 are noncoordinately upregulated by IL-6/STAT3 signaling after bile duct ligation. Lab Invest..

[23] Manabe Y, Anrather J, Kawano T, Niwa K, Zhou P, Ross ME (2004). Prostanoids, not reactive oxygen species, mediate COX-2-dependent neurotoxicity. Ann Neurol..

[24] Ahmad AS, Saleem S, Ahmad M, Dore S (2006). Prostaglandin EP1 receptor contributes to excitotoxicity and focal ischemic brain damage. Toxicol Sci..

[25] Kawano T, Anrather J, Zhou P, Park L, Wang G, Frys KA (2006). Prostaglandin E(2) EP1 receptors: downstream effectors of COX-2 neurotoxicity. Nat Med..

[26] Gendron TF, Brunette E, Tauskela JS, Morley P (2005). The dual role of prostaglandin E2 in excitotoxicity and preconditioning-induced neuroprotection. Eur J Pharm..

[27] Ahmad AS, Zhuang H, Echeveriia V, Dore S (2006). Stimulation of prostaglandin EP2 receptors prevents NMDA-induced excitotoxicity. J Neurotrauma..

[28] Ahmad AS, Ahmad M, de Brum-Fernandes AJ, Dore S (2005). Prostaglandin EP4 receptor agonist protects against acute neurotoxicity. Brain Res..

[29] Dore S (2006). GPCR antagonists as an alternative to COX-2 inhibitors: a case for PGE2 EP1 receptor. TRENDS in Pharm Sci..

[30] Ikeda-Matsuo Y, Tanji H, Narumiya S, Sasaki Y (2011). Inhibition of prostaglandin E2 EP3 receptors improves stroke injury via anti-inflammatory and anti-apoptotic mechanisms. J Neuroimmunology..

[31] Sugimoto Y, Narumiya S (2007). Prostaglandin E receptors. J Biol Chem..

[32] Abramovitz M, Adam M, Boie Y, Carriere M, Denis D, Godbout C (2000). The utilization of recombinant prostanoid receptors to determine the affinities and selectivities of prostaglandins and related analogs. Biochim Biophys Acta..

[33] Aihara E, Nomura Y, Sasaki Y, Ise F, Kita K, Takeuchi K (2007). Involvement of prostaglandin E receptor EP3 subtype in duodenal bicarbonate secretion in rats. Life Sci.

[34] Matute C, Alberdi E, Domercq M, Perez-Cerda F, Perez-Samartin A, Sanchez-Gomez MV (2001). The link between excitotoxic oligodendroglial death and demyelinating diseases. Trends Neurosci..

[35] Jones RL, Wise H, Clark R, Whiting RL, Bley KR (2006). Investigation of the prostacyclin (IP) receptor antagonist RO1138452 on isolated blood vessel and platelet preparations. Br J Pharmacol..

[36] Jiang J, Ganesh T, Du Y, Quan Y, Serrano G, Qui M (2012). Small molecule antagonist reveals seizure-induced mediatioin of neuronal injury by prostaglandin E2 receptor subtype EP2. Proc Nat Acad Sci USA.

[37] Takahashi C, Muramatsu R, Fujimura H, Mochizuki H, Yamashita T (2013). Prostacyclin promotes oligodendrocyte precursor recruitment and remyelination after spinal cord demyelination. Cell Death Dis..

